# Photovoice as a creative coping tool with the COVID-19 crisis in practical training seminar for social work students

**DOI:** 10.1177/1473325020973309

**Published:** 2021-03

**Authors:** Menny Malka

**Affiliations:** School of Social Work, Sapir College, Hof Ashkelon, Israel

**Keywords:** Photovoice, coping, social work education, Covid-19, practical training seminar

## Abstract

The Coronavirus-19 crisis has led university professors, social workers, students and social service consumers to shift to online methods of communication and teaching. In this novel, shared reality, the present paper introduces a new initiative based on implemented photovoice methodology as a tool for documenting BSW students' professional daily lives. This tool was used at a practical training seminar for 16 third year students at the School of Social Work, Sapir Academic College.

The Coronavirus-19 crisis has led university professors, social workers, students and social service consumers to shift to online methods of communication and teaching. In this novel, shared reality, the present paper introduces a new initiative based on implemented photovoice methodology as a tool for documenting BSW students' professional daily lives. This tool was used at a practical training seminar for 16 third year students^1^ at the School of Social Work, Sapir Academic College.

## Context description

The training seminar is a dedicated course, which takes place throughout the year, during which students deal with dilemmas from practical training in the field that takes place two days a week. The seminar allows students to address issues related to developing their professional identity as future social workers. By the beginning of semester two, with the outbreak of the Corona crisis, the seminar sessions became online through Zoom.

I decided to use photovoice considering my previous acquaintance with this methodology (Malka, 2020[Bibr bibr1-1473325020973309]), and particularly in view of its advantages when creating a space for dialog in which difficulties and strengths coping with a shared reality may be examined. The formation of a new online pedagogical space where creative teaching methods must be implemented also contributed to my decision to use this methodology as I prepared, in my teaching capacity, to cope with the Coronavirus crisis by attempting to combine adapted tools and methods.

## Ethical considerations

The process described has been approved by the teaching committee and ethics committee of the School of Social Work at Sapir Academic College. The students who attended the course have signed an informed consent form to approve the distribution of materials in the exhibition and/or in various scientific publications. The students whose photos are presented as examples in this article have also approved the inclusion of their full name in it, and confirmed their work description.

## Implementation of the photovoice in the training seminar

I incorporated photovoice into my training seminar sessions in the five key phases described below:

### Phase 1 – Orientation

The students were asked to create a photovoice entitled “my experience as a third-year student undergoing practical training during the Coronavirus crisis”. This phase consisted of some explanation about the methodology, photography as well as ethical principles associated with photograph-taking.

### Phase 2 – Presenting the materials and creating dialog

The outcomes, sent in advance, were compiled into a presentation viewed by the training seminar students at the beginning of the session. The presentation was followed by dialog discourse on difficulties and coping experienced in practical training during the Coronavirus crisis. Here are two examples:

#### Regression *(Figure 1)*

##### Voice (narrative)

*“This image depicts a diagram* ^2^ *of how my sessions with this patient evolved, and the regression that occurred during the tenth session, following the Coronavirus crisis. During the first sessions, his answers were brief, and no profound dialog developed. In time, our relationship was slowly built during the sessions, and progressed, so that he began to share his life prior to his incarceration with me, as well as his experience in prison, and the challenges he now faces in his daily life, following his release. Now, the situation has changed. I experienced regression during my first telephone conversation with him – it was short, and his replies were brief, as they were in our early sessions. My challenge is – how do I conduct the kind of profound discourse we had in our later sessions from a distance, over the phone?”*

In this example, Aviv described the regression that followed the shift to telephone calls, and replaced sessions held in person. Like her classmates, she too was busy coping with the decline in trust and intimacy formerly created with patients following the shift to online methods.

**Figure 1.**
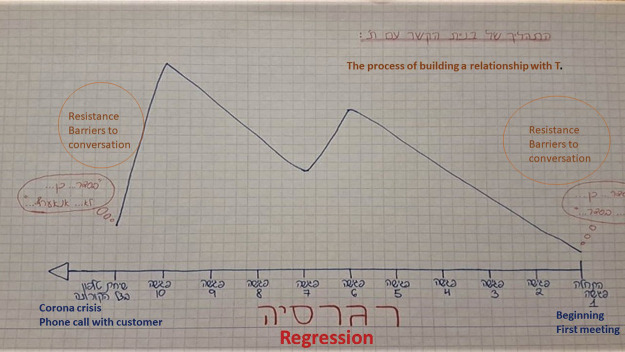
Photographed by Aviv Cohen, March 21, 2020.

#### I realized the show must go on *(Figure 2)*

##### Voice (narrative)

*“This image depicts my new ‘clinic' at home opposite a pen and paper, coffee cup, and soda water with lemon. Unlike a regular clinic, no patient sits across from me, which is a great loss of personal touch and humaneness, but also allows me to have coffee, and make notes during the session, so there are some advantages to telephone conversations. The new situation and setting have changed the relationship with those seeking my help, but I accept it because I understand that a new therapeutic contract must be drafted. This is an interesting experience for me that, at first, was very confusing, but now I am able to see its advantages.”*

In this case, Oz describes the rapid process he underwent, entitled ‘the show must go on'. This image demonstrates the new setting, and how the bachelor pad turned into a ‘clinic' due to lockdown and students' inability to travel to the training facility. His narrative prompted a discussion in the group about the new clinical setting – was it indeed acceptable to have coffee while being on the phone with a patient? And what kinds of knowledge and skills are required to conduct online therapeutic sessions?

**Figure 2.**
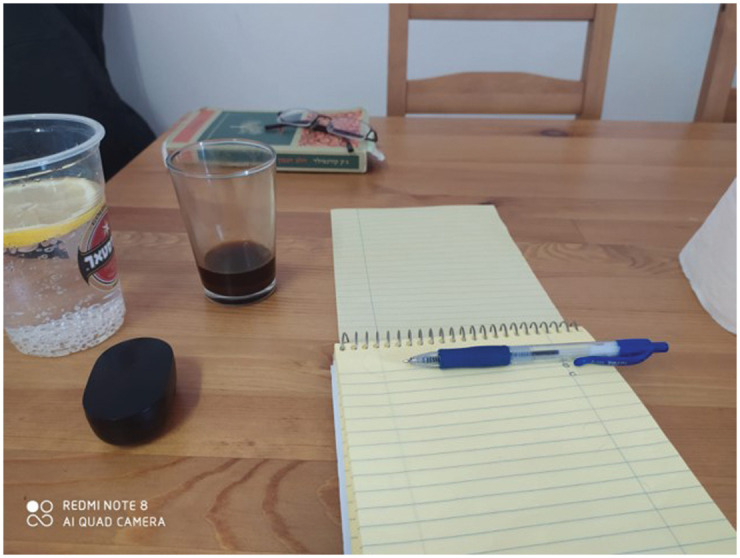
Photographed by Oz Vine, March 22, 2020.

### Phase 3 – Analyzing the dialog discourse (deconstruction)

Sixteen photovoice's were presented during the seminar sessions, enabling a dialog between participants on difficulties stemming from the shared reality they are experiencing, as well as coping methods, and new knowledge. Several key themes were identified:

1. **Challenges, questions, and concerns**: This theme consists of issues associated with gaps between the desirable and possible, concerns, how to hold onto everything with just two hands, encountering an imperfect reality, difficulties forming a relationship with patients, keeping in touch with young adults, and concerns for particularly vulnerable populations.
2. **Conceptualizing the situation**: This theme reflects the attempt to examine the new reality through existing knowledge, a (renewed) understanding of the role played by social work and workers, elements of denial and resistance to change, what online empathy means, regression processes and legitimizing fears.
3. **Coping practices**: This theme reflects coping methods reported by students, such as: shifting to online group sessions; using creativity; talking while walking; thinking outside the box; allowing oneself to experience; and continuing to work.
4. **A look to the future**: This theme deals with both the near and far future. The issues that emerged during it encouraged group participants to think about the future, place emphasis on habit, and a reality that must go on. Among the content associated with this theme were the students' upcoming graduation, weddings on the horizon, and plans for the future. These topics highlighted the importance of “the day after” and created hope for a better future.

Identifying the themes served as a process of deconstruction, which the students learned from the narrative approach (White and Epston, 1990) as well as during a qualitative research method course. This approach takes apart the new reality, experienced by each student separately, as a dominant story, i.e. as a complex and even discouraging reality, typically involving stress, uncertainty, and concerns about the future. The joint analysis allowed participants to understand that the difficult feelings shared at the beginning of the session were just a single “brick” comprising a “wall”, and that three other “bricks” are embedded within the materials they had presented: conceptualization (knowledge); coping practices, and planning the future.

### Phase 4 – Reconstruction

After the seminar session, a summary of all the topics raised was sent to the group participants, inviting them to construct a poem or story based on the narrative approach:

And *Now What?:*^3,4^

**A.**

Between realities existing and new

We realize we must switch our lens

And so, all of a sudden, the average student

Is forced to unwillingly

Understand that now that the fire has started

These our emergency times, pandemic times

But are we, like an egg in the fridge or a cake

A basic commodity with a sell-by date

It remains unclear why and how

But as we all know, here too,

“Our training must go on”

**B.**

What is the setting and therapeutic contract

When physical space becomes unimportant?

And how do we respond to silence or humming

When empathy is not easily conveyed via Zoom

How can we stop worrying?

Whether we can touch someone without touching them?!

And during a process with an elderly woman

Who, to her dismay, is inaccessible?

For we, by the book, have set up a meeting

And it is simply a matter of attitude

Nothing has been done deliberately, God forbid

She simply did not understand us online

**C.**

But here too, much like art

The new crisis provides opportunity

For it is our job to be responsible

And understand that this is creativity's time to shine

For we can despair and go around in circles

Or we can reinvent the wheel

We can run or flee

Or we can be those who think outside the box

No need to hide the difficulty or ignore it

Instead we should understand that incompleteness is now wholeness

One piece and then another like a jigsaw puzzle or Lego

Helps to overcome the regression serving the ego

And am I alright? Or is it a joke

To hold a therapeutic session while out for a walk

And suddenly, panic stealthily strikes

What about the patient whose husband is violent towards her?

Most questions remain unanswered

But right now, understanding that is important too

**D.**

In short

A training seminar conducted on my computer (Zoom)

Has left me a little confused, sitting and thinking

Asking myself out of the here and now

What a third-year student has got to do with this situation

Let me engage in some mundane moaning

About mandatory participation and tiring studies

Asking for grades to be changed and due dates extended

Trying to understand how to get more grants

Or perhaps deep down at the bottom of all those things

That are building up and coming together

An understanding begins to seep through

That it is no longer just our degree or year end

Amongst this mess and uncertainty

We are among those that can change our reality

**E.**

And to the truly important things

We must also direct our gaze

As we have a newly affianced member in our group

And perhaps that is the bottom line

It will be alright soon, and a wedding will be held

The poem written demonstrates the use of group or community forces and resources (McDonald et al., 2012) as means of rewriting the coping narrative in a process that, enables the construction of an alternative story, and allows the group to reauthor its narrative (White and Epston, 1990). The poem has five main sections: **the first** demonstrates the experience of encountering the reality typical of crises, and the changes associated with it; **the second** touches upon the challenges of practical training within such a reality, such as “online empathy”; **the third** is based on the process of transforming crisis into opportunity, centering on new knowledge developed in light of a crisis situation; **the fourth** deals with the shift between past, present, and future, to uncover where the students were then, are now, and will be in due course; and **the fifth** reminds us all of humanity's survival instinct, as the group rejoices over the news of their fellow student's engagement.

### Phase 5 – Returning the outcome to the group and disseminating it

The poem was sent to and approved by the group. In the spirit of photovoice methodology, it was subsequently disseminated in the school of social work community as an inspiring tool that provides meaning to coping with the crisis. Writing and presenting the poem also served as a vehicle for giving the experience meaning, that reflects the seminar group's coping capabilities as a whole. In effect, this was a group process that showed the seminar participants how each member's contribution created a synergy within the coping process whereby the whole – the group – becomes greater than the sum of all its parts.

## Summary

Alongside humor and pleasure, which are part of the photovoice approach, this process demonstrates the use of creativity and art by a community of social work students, for coping with crisis and disaster situations. It also shows how the forces, capabilities, creativity and bodies of knowledge learned (the narrative approach and qualitative research) may be used to tell (a chapter within) the story of that community, as well as encourage its members to reauthor and rewrite the narrative so that it provides them with control and mitigates uncertainty.

In fact, like the students, I too began online teaching with many concerns, facing an experience that was entirely new to me. The issues presented by the students through photovoice, and the ensuing discourse, greatly contributed to my own coping process as a social worker and lecturer. The way the group had joined forces to translate the initial outcome, and the unique value of the poem as a coping tool, allowing for difficulties to be recognized and various coping methods to emerge, were inspiring, encouraging me to “dream” of the next stage, and assimilate this methodology in other platforms. For instance, the success of the online session led me to start other professional initiatives, calling (as a father of two daughters with special needs and a professional in the field) for a photovoice project for special families. The students also taught me how important it was not to let the new situation terrify you, but instead to view it as an opportunity to implement current knowledge while conceptualizing the new knowledge accumulated as we worked, understanding that the post-Coronavirus world will never be as it once was.

## Notes

1 The author would like to thank the students who participated in the process described in the article and created the products that appear in it: Adi Hari; Alia Baumfeld; Aviv Cohen; David Solomon; Daniel Beker; Gal Spinard; Nirit Eli-Odaya; Noga Amrami; Oren Samama; Oz Vine; Ravit Inbar; Ruth Degorker; Shahar Ben-Moshe; Tair Amar; Yotam Ethan; Ifat Lutzky- Ze'evi.

2 In keeping with Hebrew, which is written right-to-left, this graph should also be viewed thus.

3 The student Oren Samama was in charge of collecting the themes and designing the poem.

4 The original poem in Hebrew has an AB AB rhyming scheme.
